# The Reactivity and Stability of Polyoxometalate Water Oxidation Electrocatalysts

**DOI:** 10.3390/molecules25010157

**Published:** 2019-12-31

**Authors:** Dandan Gao, Ivan Trentin, Ludwig Schwiedrzik, Leticia González, Carsten Streb

**Affiliations:** 1 Institute of Inorganic Chemistry I, Ulm University, Albert-Einstein-Allee 11, 89081 Ulm, Germany; Dandan.gao@uni-ulm.de (D.G.); ivan.trentin@uni-ulm.de (I.T.); 2 Institute of Theoretical Chemistry, Faculty of Chemistry, University of Vienna, Währinger Str. 17, 1090 Vienna, Austria; ludwig.schwiedrzik@univie.ac.at; 3 Helmholtz-Institute Ulm (HIU), Helmholtzstr. 11, 89081 Ulm, Germany

**Keywords:** polyoxometalate, water oxidation catalysis, oxygen evolution reaction, self-assembly, electrocatalysis

## Abstract

This review describes major advances in the use of functionalized molecular metal oxides (polyoxometalates, POMs) as water oxidation catalysts under electrochemical conditions. The fundamentals of POM-based water oxidation are described, together with a brief overview of general approaches to designing POM water oxidation catalysts. Next, the use of POMs for homogeneous, solution-phase water oxidation is described together with a summary of theoretical studies shedding light on the POM-WOC mechanism. This is followed by a discussion of heterogenization of POMs on electrically conductive substrates for technologically more relevant application studies. The stability of POM water oxidation catalysts is discussed, using select examples where detailed data is already available. The review finishes with an outlook on future perspectives and emerging themes in electrocatalytic polyoxometalate-based water oxidation research.

## 1. Introduction

**Figure 1:**
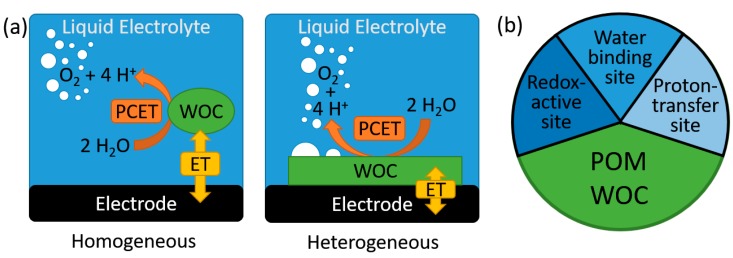
(**a**) Simplified schematic illustration of the chemical and electrochemical steps occurring during homogeneous and heterogeneous electrocatalytic water oxidation. (**b**) Illustration of the functional components required within a molecular POM-WOC. ET—electron transfer; PCET—proton coupled electron transfer; polyoxometalate (POM); water oxidation catalyst (WOC).

The splitting of water into hydrogen and oxygen is a key technology for sustainable energy conversion and storage [1,2,3]. Particularly, electrocatalytic water splitting is relevant to generate hydrogen as secondary energy carrier from intermittent energy sources, such as solar or wind power [3,4,5,6]. In general, electrocatalytic water oxidation catalyst (WOC) research can be broadly subdivided in homogenous catalysis using molecular catalysts and heterogeneous catalysis, mainly focusing on solid-state, often nanostructured materials. Note that while homogeneous WOC catalysis occurs in one phase (i.e., in solution), under electrocatalytic conditions, the role of interfacial electrochemical processes between the molecular catalyst and the electrode surface have to be considered when rationalizing reactivities and the mechanism. Thus, in homogeneous electrocatalysis, the electron transfer to the catalyst is an interfacial process between electrode and solution, while the catalytic turnover (via a proton-coupled electron transfer mechanism, PCET) is expected to occur in solution (Figure 1) [7]. In contrast, in heterogeneous catalysis, electron transfer and substrate turnover occur at the catalyst-solvent interface [3]; since water oxidation involves formation of a gaseous product, O_2_, triple-phase boundaries between solid catalyst, liquid electrolyte and gas have to be considered also [8]. In addition, effective electron transfer between the heterogeneous catalyst and the actual electrode (often metal or glassy carbon) needs to be considered to fully understand the macroscopically observed WOC activity [3,9].

Further, when technological usage is targeted, the operating conditions of modern water electrolyzers have to be considered. Currently, three main types of water electrolyzers are employed: Alkaline water electrolyzers (AWE) operate in highly alkaline aqueous solution (pH > 13), which restricts catalyst choice due to stability considerations [10]. In AWEs, NiFe layered double hydroxides are often used as WOCs with high corrosion stability [10,11]. Additionally, current research is exploring other transition metal (oxy)hydroxides as alternative WOCs [12]. In contrast, proton exchange membrane (PEM) water electrolyzers operate under acidic conditions (pH < 2) using a solid membrane for proton transport instead of liquid electrolytes [13]. This also limits catalyst selection to materials which can withstand these harsh conditions [13]. In PEM water electrolysis, typically noble metal oxides, such as IrO_2_ or RuO_2_, have been used as catalysts [14]. More recent studies have reported noble-metal free metal oxides as potential alternative WOCs in PEMs [15]. Anion exchange membrane water electrolyzers (AEMs) can operate at near pH-neutral conditions [16], so that catalyst selection is less critical, resulting in the use of various metal oxides/hydroxides as WOCs (e.g., Co_3_O_4_, CuCoO_x_) [16,17]. Note that while the technology-readiness levels of these established systems and WOCs are often in the prototype stages, for the polyoxometalate-catalysts discussed here, many fundamental questions need to be addressed before advanced upscaling and deployment can be envisaged; see the following sections.

One of the most intriguing classes of molecular WOCs are molecular metal oxide clusters, so-called polyoxometalates (POMs). POMs are anionic metal oxo clusters typically formed by pH-, temperature and concentration-dependent complex self-assembly routes in aqueous solutions [18,19,20,21,22]. POMs are typically based on early, high-valent transition metals (mainly Mo, W and V), where the outer surface of the POM anions is stabilized by unreactive, strong metal-oxo double bonds. Non-functionalized POMs are, therefore, not suitable for water oxidation, as the redox potential of the high-valent central metals (Mo(VI), W(VI), V(V)) is not sufficient for electron abstraction from water or oxo ligands. Thus, functionalization of the cluster shell with suitable reaction sites, mainly transition metals, is used as central design principle for active POM-WOCs [23,24,25,26]. There are several fundamental requirements for the structural and chemical design of viable POM-WOCs:

(1) One or several redox-active transition metals need to be incorporated in the POM framework to enable the proton-coupled, four-electron water oxidation process. The use of several metal sites facilitates this approach, as the four-electron transfer can be split up between the redox-active metal centers present. Many POM-WOCs reported feature four or more metal sites, some species featuring up to nine redox-active centers [27,28].

(2) For water oxidation to proceed, the catalysts require substrate binding sites; i.e., accessible coordination sites where aquo ligands can bind [26]. While some reports recently discussed the use of POMs without water binding sites for WOC, further studies are required to fully understand the oxygen evolution processes reported for these systems [29,30,31].

(3) The proton-coupled electron transfer reactions required for water oxidation can be facilitated by nearby proton transfer sites; i.e., Bronsted bases capable of supporting the release and transfer of protons from the water substrate. Due to their general structures, POM-WOCs typically feature terminal and bridging oxo-ligands close to the reaction sites, so that oxo-ligand-assisted proton transfer is possible. However, thus far, few details are known and the design of optimized POM structures for this purpose is still in its infancy [32]. In the following, we will describe the tremendous progress made in the field of molecular POM-WOC research with a focus on electrocatalysis under homogeneous and heterogenized conditions. We will outline major breakthroughs made, and will detail current challenges together with future directions for the field.

Broadly, the development of homogeneous POM-WOC research can be separated into two phases: pioneering initial studies in the 2004–2010 period were focused on prototype, noble-metal functionalized polyoxotungstates. Starting from 2010, more and more work appeared where 3d transition metals (mainly Co, Mn, Fe and Ni) were used as redox-active sites for water coordination and oxidation. However, the coordinative lability and fast ligand exchange rates of many 3d metal cations [33] has led to discussions in the literature concerning the stability of the respective POMs depending on the chosen reaction conditions [34]. We first review the development of the field of electrocatalytic POM-WOC research under homogeneous (Section 2) conditions; then, describe how these studies are supported by theoretical mechanistic analyses (Section 3); and then, move on to heterogeneous conditions where POM-WOCs are deposited on functional surfaces (Section 4). We then return to discussing the question of POM stability under different experimental conditions, (Section 5), and finally, provide a brief outlook at future developments from the authors’ point of view. (Section 6)

## 2. Homogeneous Electrocatalytic Water Oxidation by POMs

**Ruthenium-containing POM-WOCs:** We will start our discussion with the first examples of POM-based water oxidation studies, which were focused on ruthenium-substituted POM species. In 2004, Shannon and colleagues reported the electrochemical oxygen evolution by the di-Ru-species [Ru^III^_2_Zn_2_(H_2_O)_2_(ZnW_9_O_34_)_2_]^14−^ when performed by pulsed voltammetry in aqueous phosphate buffer at pH 8.0. The authors focused on the correlation between cluster structure and oxygen evolution activity and reported that Ru-free reference POMs and a mono-Ru-functionalized POM with a related structure did not show oxygen evolution under the conditions given. This relates nicely to earlier work, where dinuclear ruthenium complexes had been employed to drive the oxygen evolution by water oxidation [35]. The authors’ final statement, “The proximity of the two Ru atoms appears to be a key factor in the electrocatalyst’s ability to generate O_2_,” [36] summarizes the challenge for POM-WOC research; i.e., the quest for polynuclear redox-active metal-oxo aggregates stabilized by POMs capable of sustained water oxidation.

**Figure 2:**
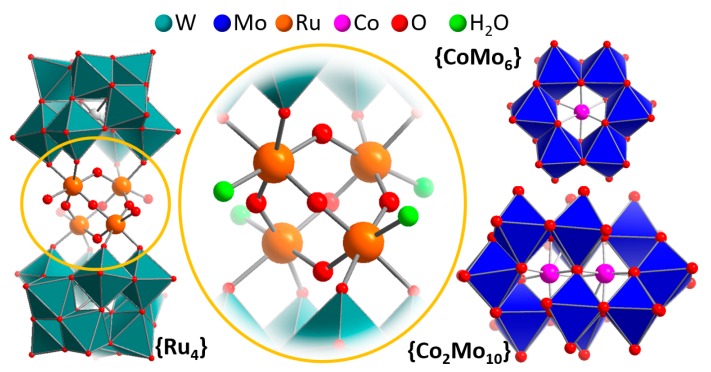
Left: Illustration of the structure of the POM-WOC **{Ru_4_}**, highlighting the central [Ru_4_O_6_] sandwiched between two polyoxotungstate ligands (teal polyhedra). Center: detailed view of the reaction site, highlighting the redox-active metal centers (orange), aquo ligands (green) and possible proton transfer sites (red). Right: Illustration of the **{CoMo_6_}** and **{Co_2_Mo_10_}** clusters.

Following that study, in 2008, the groups of Bonchio [37] and Hill [38] virtually simultaneously reported the WOC activity of the tetra-ruthenium-functionalized polyoxotungstate cluster [{Ru_4_(μ-O)_4_(μ-OH)_2_(H_2_O)_4_}(γ-SiW_10_O_36_)_2_]^10−^ (= **{Ru_4_}**). The species features a [Ru_4_O_4_] core sandwiched between two so-called lacunary Keggin anion fragments [γ-SiW_10_O_36_]^8−^, which act as inorganic tetradentate oxygen donor ligands and stabilize the ruthenium oxo core. This prototype beautifully illustrates the fundamental requirements for operational POM-WOCs outlined in Section 1: As illustrated in Figure 2, **{Ru_4_}** features four redox-active Ru(IV) reaction sites for electron transfer. Each Ru center features a terminal ligand binding site which—in the native species—is occupied by a water ligand. Further, the tungsten oxo shell of the POM offers proton transfer sites close-by to the redox-active reaction site, which could engage in Bronsted acid-base chemistry during the water oxidation process. Hill and colleagues reported the electrochemical characterization of the system and noted that under acidic conditions (pH 1 (aqueous 0.1 M HCl) and pH 4.7 (aqueous 0.4 M acetate buffer)), no catalytic current increase was observed, whereas for an aqueous 0.15 M NaCl solution at pH 7, the authors reported a redox wave at E > 0.9 V versus Ag/AgCl, which they assigned to the water oxidation by **{Ru_4_}**.

Starting from 2010, research in POM-WOC chemistry became very much focused on exploring the use of earth-abundant metals—mainly 3d metals such as Mn, Co or Ni as reaction sites in POM-WOCs, and the reader is directed to several general recent reviews describing this compound class [23,24,25,39]. Note that most of these studies focused on chemical or photochemical water oxidation studies, while electrochemical analyses of homogeneous POM-WOCs solutions were often mainly used to probe the fundamental electrochemistry and gain initial insights into a possible WOC activity. The following section will illustrate recent progress in 3d-transition metal functionalized POM-WOC research using selected examples:

**Cobalt-containing POM-WOCs:** The identification of the central “metal-oxo cubane” architecture sandwiched between two lacunary polyoxotungstates (Figure 2) led to a flurry of activity—exploring related structures for WOC. In pioneering work reported in 2010, Hill and colleagues described the homogeneous electrochemical and photochemical WOC activity of a noble-metal-free Co-tungstate catalyst, [Co_4_(H_2_O)_2_(PW_9_O_34_)_2_]^10^^–^ (=**{Co_4_}**) [40]. When deployed in aqueous phosphate buffer as electrolyte at pH 8.0, the system was reported to show a oxidative catalytic wave with onset potential of ca. 1.0 V (versus Ag/AgCl). Further studies of the system under electrochemical conditions led to some unexpected results which will be discussed in the context of catalyst stability in Section 5.

Galán-Mascarós and colleagues focused their efforts on exploring the WOC activity of high-nuclearity cobalt tungstate POMs. As an initial test system, the group explored the nona-cobalt-functionalized species {Co_9_(H_2_O)_6_(OH)_3_(HPO_4_)_2_(PW_9_O_34_)_3_}^16−^ (=**{Co_9_}**) [28]. Briefly, the structure consists of a central cobalt-oxi-hydroxide-phosphate core, {Co_9_(H_2_O)_6_(OH)_3_(HPO_4_)_2_} stabilized by three peripheral lacunary [PW_9_O_34_]^9−^ anions. The authors performed initial electrochemical experiments on aqueous **{Co_9_}** solutions and observed sustained oxidative currents. However, more in-depth studies showed some intriguing effects, so more details of this system are discussed in Section 5.

**Ni-based POM-WOCs:** Initial insights into Ni-containing tungstate WOCs were reported by Ding and colleagues who explored the oxidative activity of the di-nickel species [{β-SiNi_2_W_10_O_36_(OH)_2_(H_2_O)}_2_]^12−^ (=**{Ni_2_}**). Electrochemical studies of **{Ni_2_}** and the reference NiSO_4_ in aqueous borate buffer (80 mM, pH 9.0) showed notably different cyclic voltammetry: while **{Ni_2_}** showed a catalytic oxidative wave with onset at ≈1.0 V (all potentials given versus Ag/AgCl), the reference showed a first oxidative wave at ≈0.9 V, followed by the onset of a catalytic wave at ≈1.0 V [41]. The authors explored the possible leaching of Ni^2+^ from the cluster using EDTA as a chelating agent, which inhibits WOC activity. While for the NiSO_4_ reference, significant loss of catalytic activity was observed, **{Ni_2_}** showed no significant changes to its electrocatalytic WOC activity in the presence of EDTA, suggesting that a molecular, homogeneous WOC catalyst is present under the conditions studied. Further, no catalytically active, heterogeneous nickel oxide depositions were found on the electrode after water oxidation by **{Ni_2_}**, excluding the possibility of a solid-state catalyst formation [41].

In addition to the sandwich-type tungstates discussed above as homogeneous WOCs, there is a related class of transition metal functionalized POMs with reported water oxidation activity, that is, POMs, for which the transition metal is incorporated as central internal template (Figure 2). Prime examples were reported by Sakai and colleagues, who used the species [H_6_Co^III^Mo_6_O_24_H_6_]^3−^ (**{CoMo_6_}**) and [H_4_Co^III^_2_Mo_10_O_38_]^6−^ (**{Co_2_Mo_10_}**) as homogeneous, light-driven WOCs [42], and by Das and colleagues who explored [Co^II^W_12_O_40_]^6−^ embedded in the metal organic framework ZIF-8 as electrochemical WOCs [31]. Remarkably, both systems show significant oxygen evolution activity while the expected cobalt reaction sites are “locked” within the POM framework and are not accessible for the binding of external water ligands. While for the cobalt molybdate systems, initial electrochemical data is required to rationalize possible water oxidation pathways, this data is available for the co-tungstate [Co^II^W_12_O_40_]^6−^. Intriguingly, the authors noticed that under homogeneous electrochemical conditions (aqueous Na_2_SO_4_ (0.1 M), pH 1.9, adjusted with H_2_SO_4_, all potentials given versus normal hydrogen electrode (NHE)), [Co^II^W_12_O_40_]^6−^ only shows one quasi-reversible redox-wave (E_1/2_ ≈ 1.1 V, assigned as Co^III/II^) within the potential window studied (0.2–1.5 V). In contrast, when drop-cast on a glassy carbon electrode, the heterogeneous [Co^II^W_12_O_40_]^6−^@ZIF-8 shows electrocatalytic oxygen evolution activity with an onset potential of ≈1.2 V at pH 1.9, and ≈1.0 V at pH 7. These preliminary results clearly show that POMs with internal redox-active transition metal sites are unexpected, but reactive components, which play a role in electrochemical and photochemical oxygen evolution, and most probably feature oxygen evolution mechanisms which are distinct from their multi-transition-metal-containing relatives [43,44].

**Figure 3:**
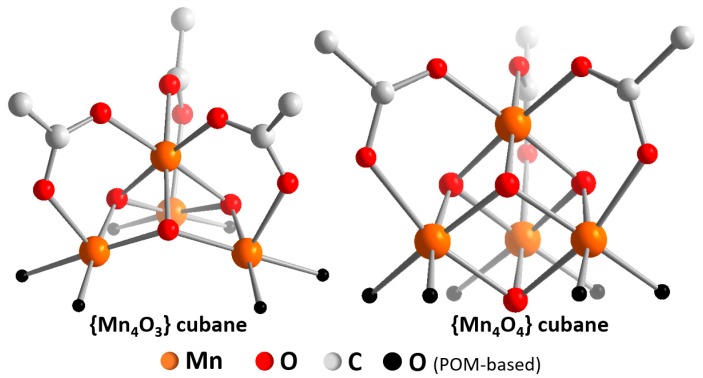
Illustration of the “defective” **{Mn_4_O_3_}** cubane core (left) [47] and the “complete” **{Mn_4_O_4_}** cubane core (right) [46]. Note that both cubanes do not feature water binding sites but are saturated by peripheral acetate ligands.

**Manganese-containing POM-WOCs:** The first study of a manganese-containing POM-WOC was reported by Scandola, Kortz, Bonchio and colleagues in 2014. The authors explored the unusual manganese tungstate [Mn_4_O_3_(AcO)_3_(A-a-SiW_9_O_34_)]^6−^ (= **{Mn_4_W_9_}**), where a defective manganese oxo cubane {Mn^III^_3_Mn^IV^O_3_} (which was missing one oxo “corner”) was stabilized by a trilacunary tungstate and three acetate ligands. The manganese oxidation states correspond to the “S_2_”-state in the Kok-cycle of natural photosynthesis [45]. Preliminary electrochemical studies of **{Mn_4_W_9_}** (aqueous Na_2_SiF_6_/NaHCO_3_ buffer, 0.5 M of Na_2_SO_4_ as electrolyte, pH 5.2) showed a pre-catalytic oxidative wave at ≈0.85 V (all potentials given versus Ag/AgCl) which was most likely due to the Mn^IV/III^ redox-couple. At a potential of ≈1.2 V, the onset of a catalytic oxidation was observed. This suggests that the WOC-active species operates from an {Mn^IV^_4_} oxidation state, in line with the current interpretation of the “S_3_”-state of the oxygen evolving complex, OEC [45]. Intriguingly, in 2016, Streb and colleagues reported a related manganese POM-WOC, [Mn_4_V_4_O_17_(AcO)_3_]^3−^ (= **{Mn_4_V_4_}**), where a “complete” Mn-oxo cubane, {Mn^III^_2_Mn^IV^_2_O_4_} was stabilized by a tetranuclear vanadate cluster and three acetate ligands [46] (Figure 3). In contrast to **{Mn_4_W_9_}**, this species was isolated as organo-soluble tetra-*n*-butylammonium salt so that electrochemistry under inert conditions was directly possible. CV in dry MeCN (containing 0.1 M *n-*Bu_4_NPF_6_ as electrolyte) showed two oxidative quasi-reversible processes (at ≈0.1 V and 1.1 V versus Fc^+^/Fc), which were assigned to Mn^IV/III^ redox couples. In the presence of water (9:1 MeCN:H_2_O mixture containing 0.1 M *n-*Bu_4_NPF_6_ as electrolyte), two oxidative processes (at 0.2 V and 0.8 V versus Fc^+^/Fc) were observed together with a catalytic oxidative wave (onset potential ≈0.9 V).

Further electrochemical analyses, e.g., in the absence of water, could help to shed more light on the mechanism of water binding, water oxidation and oxygen-oxygen bond formation [48]. Of particular interest for both cubane structures is the role of the acetate ligands during Mn-oxidation, water binding and water oxidation, and a combination of experimental (spectro-)electrochemical data together with computational analyses could help to rationalize whether both systems follow similar water oxidation mechanisms, and indeed, whether they could also be models to further explore the function of the natural OEC system. Intriguingly, the original study on {Mn_4_V_4_} reported preliminary ESI mass spectrometric data, which showed a one-electron-oxidized species with one acetate ligand removed (i.e., [Mn^III^Mn^IV^_3_V_4_O_17_(AcO)_2_]^−^), which hints at the possibility that acetate removal and/or oxidation can lead the formation of accessible water binding sites on the [Mn_4_O_4_] cubane [46].

## 3. Theoretical Simulations for Mechanistic Insights into POM-WOCs

In recent years, theory has started to play an ever-increasing role in studying the reactivities of POM-WOCs. Using a variety of theoretical methods, researchers have done everything—from filling in gaps in experimental data, e.g., optimizing structures that could not be determined using X-ray diffraction [49] or aiding in the interpretation of spectroscopic [50] and electrochemical data [51]—to uncovering the mechanistic details of the entire water oxidation cycle for a given WOC [52]. The ultimate goal of these studies is the rational design of improved WOCs, using the mechanistic insight gained by theory to guide additional experimental efforts.

Early studies by Hill and colleagues [53] focused on [Ru_2_(μ-OH)_2_(H_2_O)_2_(SiW_10_O_36_)]^4−^. They concluded that the complex was incapable of catalyzing water oxidation, proposing instead that it might form the known WOC **{Ru_4_}** through a dimerization-like process. The structural and electronic properties of **{Ru_4_}** and its first five oxidation states S_0_ through S_4_ were characterized in a follow-up study [54]. It should be noted that both these studies were carried out entirely using density functional theory (DFT), by far the most widely used theoretical method in POM-WOC research. In contrast, Llobet, Bo, Bonchio and colleagues [50] carried out a combined experimental and theoretical study in which they characterized the activation mechanism of **{Ru_4_}**, finding that the S_4_ state contained four Ru^V^-OH groups open to nucleophilic attack by H_2_O. They suggested that the formation of O_2_ proceed from the S_4_ state through a complex sequence of proton and electron transfers.

**Figure 4:**
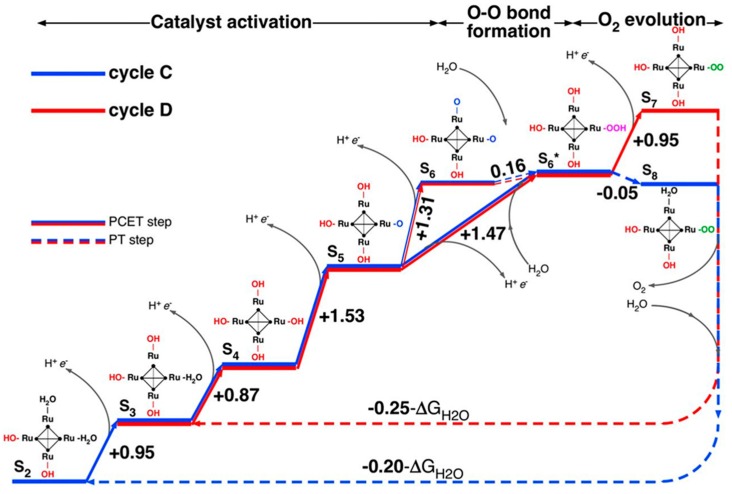
Free energy diagram of the proposed water oxidation cycles C and D involving high oxidation states of **{Ru_4_}**. Free energy differences given in eV; ΔG_H2O_ is the free energy change for the reaction 2H_2_O ⟶ O_2_ + 2H_2_ at pH = 0 and room temperature. Reproduced with permission from [56]; copyright 2013 National Academy of Science of the U.S.A.

Piccinin and Fabris [55] were the first to attempt to elucidate the entire water oxidation cycle of **{Ru_4_}** using solely theoretical methods. They initially studied a proposed mechanism wherein **{Ru_4_}** is oxidized from the S_0_ (Ru^IV^_4_) to the S_4_ (Ru^V^_4_) state in four PCET steps, to then promote water oxidation and return directly to the initial S_0_ state. However, they found that the computed free energy difference between S_0_ and S_4_ was 0.9 eV below the thermodynamic limit for water oxidation, thus, showing that the proposed S_0_–S_4_ mechanism was thermodynamically infeasible. The authors went on to show, in a joint study with Bonchio and co-workers [56], that higher oxidation states of **{Ru_4_}** play a crucial role in the water oxidation cycle, resulting in the two reactions cycles C and D, shown in Figure 4. In cycle C, **{Ru_4_}** starts in the S_2_ (Ru^IV^_2_ Ru^V^_2_) state, proceeding through a series of PCET steps to S_6_ (Ru^V^_2_ Ru^VI^_2_), followed by nucleophilic attack by H_2_O, O_2_ evolution and a return to the S_2_ state. Cycle D begins in the S_3_ state and reaches the S_7_, showing great mechanistic similarity to heterogenous water oxidation catalysis on a RuO_2_ surface. Finally, Piccinin and Fabris carried out a comprehensive theoretical investigation of the energetics of the water oxidation cycles of **{Ru_4_}** and of [Ru(H_2_O)(SiW_11_O_39_)]^5−^ [57]. They found that the overpotential of the two species is determined primarily by the oxidation state of the metal center(s) and that, while both species display similar energetics, solvent effects play a much more important role for [Ru(H_2_O)(SiW_11_O_39_)]^5−^ than for **{Ru_4_}**.

More recently, theoretical methods have also been used to study Co and Mn-based POM-WOCs. Musaev, Carbo, Poblet and colleagues [32] used DFT to investigate the water oxidation cycles of two WOCs, **{Co_4_}** and [Co_4_(H_2_O)_2_(VW_9_O_34_)_2_]^10−^. They proposed a detailed mechanism for both species, which consists of an electron transfer, a proton transfer and one PCET step—leading to the active S_2_ state; then, nucleophilic attack by H_2_O, the rate-determining step and two further PCET steps to yield O_2_. The authors also linked the experimentally observed higher catalytic activity of [Co_4_(H_2_O)_2_(VW_9_O_34_)_2_]^10−^ compared to **{Co_4_}** to increased orbital coupling between the d orbitals of V and Co. Yan, Lang and colleagues [58] have employed DFT to study the water oxidation cycle of [Mn_3_(H_2_O)_3_(SbW_9_O_33_)_2_]^12−^, also finding that nucleophilic attack by H_2_O is the rate-determining step. Likewise, the water oxidation cycle of [Mn(H_2_O)GeW_11_O_39_]^5−^ was investigated in detail [59], describing two possible mechanisms, via nucleophilic attack by H_2_O or via intramolecular O–O bonding, respectively.

Beyond mechanistic studies in gas phase or solution, some researchers have undertaken investigations into the complex interactions of POM-WOCs bound to the surfaces of a variety of materials. In this context, Piccin, Fabris and colleagues carried out groundbreaking work on the binding of **{Ru_4_}** to a functionalized graphene electrode surface, studying the mode of binding and the influence of the graphene surface on the water oxidation mechanism using DFT and molecular dynamics [60]. Nam, Kim and colleagues have studied the linker-free binding of **{Co_4_}** to N-doped carbon nanotubes using a combination of experimental and theoretical techniques [61]. Fontecave, Mellot-Draznieks, Dolbecq and colleagues investigated the immobilization of **{Co_4_}** in a metal-organic framework along with a photosensitizer, again using a combined experimental and theoretical approach [62]. Finally, Lee, Ryo and colleagues reported the synthesis of efficient WOC nanoparticles of amorphous CoWO_4_ through heat treatment of **{Co_4_}**, whose high catalytic activity could be explained with the help of DFT calculations [63].

To sum up, we have seen that theoretical methods are capable of handling a wide variety of problems in the study of POM-WOCs, demonstrating that fundamental insights into the mechanistic details of water oxidation on POM catalysts that can be gained through their applications. It appears that DFT is the method of choice, as with few exceptions, all mechanistic studies presented here adopt hybrid functionals [32,49,51,52,53,55,56,57,58,59] and implicit solvent models [32,49,50,51,52,53,54,57,58,59] for their simulations. For a more comprehensive overview of theoretical methods used in POM research, the reader is referred to the excellent review by López et al. [64] Considerable progress has been made in elucidating the water oxidation cycles of some WOCs in great detail, especially those of **{Ru_4_}** and **{Co_4_}**. However, much work remains to be done, especially outside the realm of Ru-based POM-WOCs.

## 4. Heterogeneous Electrocatalytic Water Oxidation by POMs

As discussed above, homogeneous POM-WOCs offer significant advantages, including the ability to interact with other species in solution, the catalytic involvement of each single POM anion and the possibility to analyze mechanistic details on the molecular level [65,66]. However, for technological water oxidation (as part of a full water splitting system), current technologies focus on water electrolysis. This requires the stable physical and electrical linkage between POM-WOC and suitable conductive substrates, leading to composite electrocatalysts [67]. Thus, a variety of POM-WOCs have been immobilized on conductive supports. The major approaches and substrate classes are described in the following. Note that for technological deployment at high oxidative currents and high current densities, oxidatively sensitive electrodes such as carbon-based systems could be problematic. This point is a matter of current investigation [68,69], and efforts are being made to prevent carbon degradation by surface passivation routes [70].

**Carbon paste (CP) supports:** The incorporation of electroactive compounds into carbon paste is an established approach to custom-made electrochemical working electrodes and has been explored for the electrode-immobilization of POM-WOCs as well: In 2010, Shan and colleagues reported the modification of a CP electrode with the POM-WOC [Hpy]_2_{[Co(4,40-Hbpy)_2_(H_2_O)_2_][SiCoW_11_O_39_]} in acidic solution (pH 4.5) [71]. The POM was incorporated into the CP by physical mixing/grinding of graphite powder with different amounts of the POM. The resulting paste was electrically contacted with a copper electrode and tested for electrocatalytic water oxidation in 0.5 M sodium acetate buffer (pH 4.5) containing [Ru(bpy)_3_]^2+^ (1 mM) as the redox-probe. Initial electrochemical analyses showed that the onset potentials of oxygen evolution for the modified electrode decreased compared to the POM-free reference, and the oxidative currents increased with the increasing mass loading of the POM-WOC.

A similar approach was used by Galán-Mascarós and colleagues, who integrated the cesium-potassium salt of **{Co_9_}** (**Cs_15_K{Co_9_}**) into a CP electrode at **Cs_15_K{Co_9_}** loadings between 1 and 60 wt-% [72]. The group analyzed the electrocatalytic oxidative performance of the system in a pH 7 aqueous sodium phosphate buffer (NaPi, 50 mM) with NaNO_3_ (1 M) as an electrolyte. The CV showed a strong catalytic oxidative wave at potentials of ≈1.3 V (versus NHE) and determination of the OER faradic efficiency gave values >90%. The best performances, i.e., lowest overpotentials (540 mV at *j* = 1 mA/cm^2^) and lowest Tafel slopes (95 mV/dec), were observed at POM-WOC loadings of 14%. The authors report remarkable long-term OER stability (>8 h) across a wide pH range (pH = 1–10), which is notable, as many metal oxide catalysts (e.g., RuO_2_) are labile in acidic media [72]. Post-catalytic analyses of the electrode using IR spectroscopy, SEM-EDX analyses and powder XRD analyses show that the main features of the original sample were maintained, and no indication of the formation of solid-state cobalt oxide/hydroxide phases was observed.

The group extended this work by studying the role of the counter-cation [73] using the barium-containing species Ba_8_[Co_9_(H_2_O)_6_(OH)_3_(HPO_4_)_2_[PW_9_O_34_]_3_]·55H_2_O (=**Ba{Co_9_}**), which was also incorporated into a CP electrode [27]. The resulting composite electrocatalyst was reported to outperform the state-of-the-art IrO_2_ reference even at highly acidic media (pH < 1), where catalytic activity and stability are often hampered due to the highly corrosive environment [74]. Post-electrocatalytic ICP analysis gave no indication of cobalt oxide/hydroxide formation, suggesting that the POM structure was retained [75,76]. In addition, the authors reported remarkable stability of the system and observed stable chronoamperometric current densities over a period of 24 h. Surface-sensitive techniques (Raman and XPS) and powder XRD were employed and showed no chemical or structural degradation or leaching of the catalyst after catalysis. The authors suggest that the hydrophobic environment created by the carbon-paste electrode (which contains hydrocarbons as binder) can improve the stability and activity of metal-oxide catalysts in acidic media.

**Multiwalled carbon nanotube (MWCNT) supports:** Prato, Bonchio and colleagues have explored the use of cationically functionalized MWCNTs as electrically conductive supports for the **{Ru_4_}** POM-WOC [77,78]. To this end, the authors covalently attached polyamidoamine (PAMAM) dendrimers to MWCNTs to generate cationic binding sites for **{Ru_4_}**. Deposition of the resulting composite on conductive transparent oxide ITO electrodes gave an electrocatalytic water oxidation system which was tested in aqueous phosphate buffer electrolyte (0.1 M, pH 7). The authors reported an initial oxidation wave at 0.9 V (all potentials reported versus Ag/AgCl), followed by the onset of a catalytic wave at voltages >1.10 V. Further studies showed high stability of the system at potentials up to 1.4 V. These findings were supported by scanning electrochemical microscopy (SECM) where no significant changes of the material were observed over several oxidative cycles.

**Layer-by-layer electrode assembly:** Bonchio, McCormac and colleagues have developed layer-by-layer (LbL) assembly protocols to deposit **{Ru_4_}** on indium tin oxide (ITO) and glassy carbon (GC) electrodes [79]. The method uses three alternating layers starting with the deposition of a cationic conductive polymer (PDDA, poly(diallyldimethylammonium chloride), for optimum interfacial contact with the ITO support), followed by anionic **{Ru_4_}** POM and finished with a cationic Ru-metallodendrimer photosensitizer (**[RuDend]^8+^**). Repeated deposition of anionic **{Ru_4_}** and cationic **[RuDend]^8+^** resulted in multilayer assemblies. The team explored the water oxidation performance of a six-**Ru_4_POM-[RuDend]^8+^** multilayer assembly in aqueous sodium phosphate buffer (PBS, pH 7), which gave oxidation waves at lower potential and higher oxidation current compared to reference electrodes; i.e., **{Ru_4_}** paste coated on GC and LbL-assembled eletrodes using [P_2_W_18_O_62_]^6−^ instead of **{Ru_4_}**.

**Graphene-functionalized electrodes:** Graphene and graphene-derivatives have received tremendous attention as functional conductive substrates, and they have also been employed as supports for POM-WOCs [80]. One significant advantage is that catalyst deposition on graphene is often possible without using additional binders, such as polymers [81,82,83]. In a pioneering example, Bond, Hill and colleagues used a modified graphene-based electrode for anchoring **{Ru_4_**} [84]. To this end, the authors electrochemically deposited graphene on GC or ITO electrodes under wet-chemical conditions. Subsequently, the graphene surface was functionalized with **{Ru_4_}** (as Rb_8_K_2_**{Ru_4_}** salt) by simple overnight immersion into a suitable POM solution. The group assessed the WOC performance of the composite electrodes in 0.1 M aqueous sodium borate or phosphate buffer (pH 7.5) and explored reactivity changes in the presence or absence of additional alkali and alkali earth nitrates as supporting electrolytes (Li^+^, Na^+^, K^+^, Mg^2+^, Ca^2+^). The authors noted significant reactivity differences depending on the chosen electrocatalytic conditions; optimum reactivity was observed for borate buffered solution in the presence of 1 M Ca(NO_3_)_2_. This highlights the critical impact of “secondary” reaction parameters which can have a significant influence on the technological applicability of the materials developed [77]. The authors also explored the post-catalytic stability of the electrode using chronoamperometric analyses together with SEM-EDX studies and noted no significant structural or chemical changes.

**Mesoporous carbon nitride (MCN) support:** MCN is a promising candidate for a POM-WOC support, as it combines high porosity with high conductivity and anchoring groups (e.g., –NH and –NH_2_) suitable for POM immobilization. Pioneering studies by Wu and colleagues reported the deposition of **{Co_4_}** on MCN using a vacuum-assisted impregnation route [85]. The method involved the vacuum-assisted removal of volatile pore-contents from protonated MCN (containing –NH_3_^+^ groups), so that subsequent immersion in an aqueous **{Co_4_}** solution resulted in optimized catalyst uptake into the pores. The composite was drop-cast on ITO substrates and used as water oxidation anode in aqueous phosphate buffer (pH 7). Compared with reference systems, the electrode showed higher current densities and earlier onset potentials together with high faradaic efficiency (*≈*100%). Post-catalytic analyses, including UV/Vis spectroscopy and powder XRD, suggested a high stability of the structure and composition of the composite. Mechanistic analyses of the catalyst performance using X-ray absorption near edge structure (XANES) spectroscopy showed that the interactions between **{Co_4_}** and MCN were contributing to the observed WOC activity.

**Metal foam support:** For technological applications, high surface area metal electrodes, e.g., commercially available metal foams, are ideal catalyst supports. However, the chemically and mechanically stable linkage between POM-WOC and metal surface is still challenging [86]. Streb, Song and co-workers have explored a facile one-step hydrothermal reaction to deposit microcrystals of a Co-Ni tungstate POM-WOC ([Co_6.8_Ni_1.2_W_12_O_42_(OH)_4_(H_2_O)_8_]) on nickel foam using Na_3_[PW_12_O_40_] and Co^2+^ as molecular precursors [87]. The resulting electrode was used for water oxidation and showed sustained oxidative activity in alkaline aqueous electrolyte (0.1 M KOH, pH 13) with a low overpotential (360 mV at 10 mA/cm^2^), low Tafel slope (126 mV × dec^−1^) and high faradaic efficiency (96 ± 5%). Chronoamperometry over 10 h confirmed the long-term stability of the composite electrode, while post-catalytic analyses showed no significant structural changes of the system. Remarkably, this is the first report of a POM-based water oxidation electrode which was stable under highly alkaline conditions (pH 13), wherein most POMs typically hydrolyze within a few minutes.

**Zeolitic imidazolate framework (ZIF) supports:** As described above, pioneering studies have used ZIFs for the immobilization of the POM-WOC [CoW_12_O_40_]^6−^ (see Section 2) [31]. The resulting POM-WOC@ZIF composite was coated on glassy carbon electrodes for water oxidation studies in aqueous 0.1 M Na_2_SO_4_ (pH 8). In addition to the results discussed above, chronoamperometry showed that the catalytic stability was sustained for at least 8 h (at *E* = 1.2 V versus NHE) and the initial activity was retained even after 1000 CV cycles between 0.2 V and 1.2 V versus NHE). Further, the powder XRD patterns of the post-catalytic samples were virtually unchanged from the native system, and no leaching of Co^2+^ into the solvent was observed by ICP. This suggests that the system combines high activity with stability, so that more research can be directed into the intricacies of the interactions between ZIF support and POM.

## 5. Stability of POM-WOCs under Catalytic Operation

As outlined above, technological deployment of catalytic water splitting requires catalysts which are fast, selective, stable and economically viable. This has made POMs prime candidates for WOC reactions, as they combine high activity [40] with facile synthetic access based on oxidatively robust, earth-abundant components [46]. However, with respect to the long-term stability of POM-WOCs under catalytic turnover conditions, several systems have been debated in the literature, and particularly, Co-POM-WOCs have been analyzed under a variety of conditions to explore their (in)stabilities [22]. The ongoing debate is summarized in a quote by one of the protagonists in the field, Richard Finke: “Indeed, the hypothesis has been advanced that Co-POMs are a (if not the) superior class of robust WOCatalysts [40,88]. However, an important alternative hypothesis has also appeared: namely, that no Co-POM is sufficiently stable in water to be a long-lived WOCatalyst,” [89]. This section, therefore, explores the current knowledge for two model POM-WOC classes, i.e., Co and Mn-functionalized POM-WOCs, and explores their stability based on the most up-to-date data available. These systems were chosen for their technological importance, but also because both cobalt and manganese solid-state oxides or colloids are known WOCs, and distinguishing between one of their molecular POMs and its degradation products is often non-trivial.

**{Co_4_}:** As described in Section 2, **{Co_4_}** was the first example of a Co-based POM-WOC, and consequently, received widespread interest [1]. The authors of the study discuss that the synthetic approach to **{Co_4_}** could enable a self-repair mechanism [90]. To verify the stability of the POM-WOC under the given reaction conditions and distinguish its reactivity from possible degradation products (Co(II) ions, Co-hydroxide CoO_x_ phosphate [91]), a number of experimental analyses were performed: first, the authors verified compound stability over 1 month in aqueous phosphate buffer (30 mM, pH = 8) using UV–Vis- and ^31^P-NMR-spectroscopy. In addition, post-catalytic ^31^P-NMR studies showed no significant changes from the pre-catalytic reference spectra. The authors also examined **{Co_4_}** stability at different pH values (between 3 and 9) by ^31^P-NMR and noted no degradation after 24 h. Comparative WOC poisoning experiments were performed, comparing the reactivity loss for Co(II) ions with **{Co_4_}**, using 2,2-bipyridine (bpy) as the chelating poison. While a drastic loss of reactivity was observed for the Co(II), only a minor drop in reactivity was observed for **{Co_4_}.** Further aged Co(II) solutions showed significant reduction in reactivity after 3 days, while no similar effects were observed for identical **{Co_4_}** solutions.

Shortly after the initial publication of the POM-WOC-activity of **{Co_4_}**, Finke and colleagues investigated **{Co_4_}** stability for electrocatalytic WOC in aqueous sodium phosphate buffer at pH 8.0 using a glassy carbon electrode [76]. Initial analyses using UV–Vis-spectroscopy showed notable spectral changes under identical conditions, as described in the original publication, after only 3 h of “aging.” This observation was supported by electrochemical analyses of the system, where methods including modified stripping voltammetry [92] and calibrated linear sweep voltammetry verified the formation of “free” Co(II) in solution.

**Figure 5:**
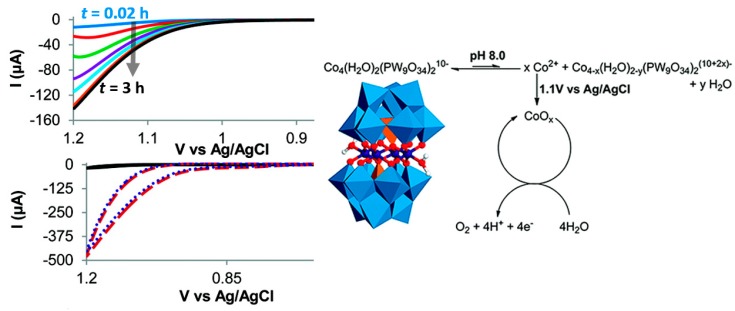
Left top: time-dependent LSV curves of **{Co_4_}** (0.5 mM) in aqueous sodium phosphate buffer (pH 8.0). Left bottom: CV scans of of **{Co_4_}** (0.5 mM) in aqueous sodium phosphate buffer (pH 8.0) immediately after dissolving (black), after 30 min electrolysis at 1.1 V versus Ag/AgCl (red dashed) and of the rinsed electrode after 30 min electrolysis at 1.1 V versus Ag/AgCl, immersed in a pure aqueous sodium phosphate buffer (pH 8.0, blue dotted). Adapted from [76] with permission; copyright 2011, American Chemical Society.

The most compelling evidence for the degradation of **{Co_4_}** under electrochemical conditions (which were different to the chemical oxidation conditions reported in the original paper [40]!) was the observation of Nocera-type Co oxide phosphate on the glassy carbon electrode [93]. This reactivity was observed upon constant potential electrolysis of a phosphate-buffered **{Co_4_}** solution (pH = 8, *E* = 1.1 V versus Ag/AgCl, *t* = 30 min). Scanning electron microscopy/energy dispersive X-ray spectroscopy (SEM-EDX) confirmed the Co-oxide-phosphate film formation on the glassy carbon electrode, and subsequent CV studies where the electrode was rinsed and transferred into a new (i.e., POM-free) electrolyte solution showed that the catalytic activity was retained. This data strongly suggested—under the given conditions—the dominant catalyst is heterogeneous Co-oxide-phosphate [91], while the POM acts as a pre-catalyst, as shown in the Figure 5. In summary, the use of highly oxidative electrochemical conditions together with the presence of phosphate buffered solutions seem to be major contributors to the **{Co_4_}** degradation and formation of solid-state, amorphous, Co-oxide-phosphate (often called CoPi) WOCs [93]. Note that extensive work, mainly by Nocera and colleagues, has demonstrated that CoPi-systems are efficient, long-lived and “self-healing” water oxidation electrocatalysts [94].

Active research into the diverging stability and reactivity of **{Co_4_}** was still ongoing when this review was prepared [40,76,95,96,97]. From the authors’ perspective, current studies suggest that the exact reaction conditions, including being an electrochemical or photochemical reaction, types of solvent, electrolyte salt and buffer, concentrations of the catalyst and the buffers and type of electrodes used, could all affect the stability and reactivity reported. Thus, from our perspective, the main lessons for researchers in the field are that (a) a wide range of stability/reactivity analyses are required to assess whether the system under investigation is a homogeneous POM-WOC or a heterogeneous degradation/rearrangement product thereof; (b) observations made under a specific set of experimental conditions hold little value once the experimental conditions are changed, and the whole set of stability/reactivity analyses need to be deployed again to verify the POM integrity. For more details, the reader is referred two recent in-depth analyses of the matter [34,97].

**{Co_4_V_2_}:** Following their original **{Co_4_}** publication, Hill and co-workers later reported the enhanced WOC activity of a derivative species, [Co_4_(H_2_O)_2_(VW_9_O_34_)_2_]^10−^ (= **{Co_4_V_2_}**), where the central phosphate present in **{Co_4_}** is replaced by a [VO_4_]^3−^ template [88]. Density functional theory (DFT) calculations suggested that the higher WOC activity might be related to the presence of the vanadium (V) atoms, which modulated the electronic structure (and thus, electron transfer capabilities) of the species; see Section 3. The authors explored the stability of **{Co_4_V_2_}** in aqueous borate buffer at pH = 9 and reported no changes of the UV–Vis spectrum over 24 h, and the retention of a characteristic ^51^V-NMR signal (at −506.8 ppm) over 1 month. Further photochemical, light scattering and other techniques, according to the authors, also supported the stability of **{Co_4_V_4_}** during catalysis.

However, sometime after the original **{Co_4_V_2_}** publication, Finke and co-workers reported deviating results regarding the stability of the cluster under water oxidation conditions. First, the group analyzed the synthetic procedures reported for **{Co_4_V_2_}** in the literature and demonstrated that under typical synthetic conditions, significant amounts of *cis-*[V_2_W_4_O_19_]^4−^ are formed as impurities [98]. The authors suggested that this impurity, rather than **{Co_4_V_2_},** accounts for the unchanging ^51^V-NMR signal at −506.8 ppm, and thus cannot be used as a proxy for the stability of the cobalt-containing POM. The authors also used a ^31^P-NMR-based line-broadening method reported earlier by Nocera and colleagues [99,100] to assess the amount of “free” paramagnetic Co(II) in solution. Their results suggest that after only a few minutes of reaction time, half of the total Co(II) in solution is present as “free” non-cluster-bound Co(II), while their results indicate that after 1 h, all Co(II) has been released from the cluster (observed at pH 5.8 and 8.0). Further evidence for Co(II) release was obtained by the electrochemical adsorptive cathodic stripping method [76], during which “free” Co(II) is first complexed by dimethyl glyoxime and subsequently adsorbed on a metal film by cathodic stripping. The results indicated the full release of Co(II) from the **{Co_4_V_2_}** POM in solutions buffered at value pH = 8.0 and 9.0 after three hours of aging. Furthermore, constant potential electrolysis (at 1.1 V versus Ag/AgCl) of **{Co_4_V_2_}** solutions buffered at pH = 5.8, 8.0 and 9.0 all resulted in the deposition of cobalt-oxide-phosphate films, as already seen for the original **{Co_4_}** samples.

**{Mn_4_V_4_}:** As discussed in Section 2, **{Mn_4_V_4_}** is a model of the oxygen evolving complex OEC in Photosystem II. The catalyst performs WOC under homogeneous, light-driven conditions in water-containing organic solvents; e.g., acetonitrile:H_2_O 9:1, *v*:*v* mixtures [46]. For functional relevance to the OEC, studies under aqueous conditions would, however, be most interesting. In a follow-up study, the authors explored the reactivity of **{Mn_4_V_4_}** under aqueous conditions. Due to the lipophilic *n-*Bu_4_N^+^ cation, water solubility of the compound is very low, so that prolonged sonication was required to obtain a clear, seemingly homogeneous solution. Analysis of the sample using dynamic light scattering, however, showed the formation of colloidal particles in the ≈90 nm range. Further studies using elemental analysis, electron microscopy and spectroscopic methods showed that the particles are composed of amorphous solid-state manganese vanadium oxide of the approximate composition VMn_5_O_10_. Interestingly, the spherical particles show water oxidation activity when exposed to chemical oxidants, such as cerium (IV) ammonium nitrate, highlighting that care has to be taken when considering POM-WOC degradation, as the degradation products might very well still be active WOCs [101].

**Figure 6:**
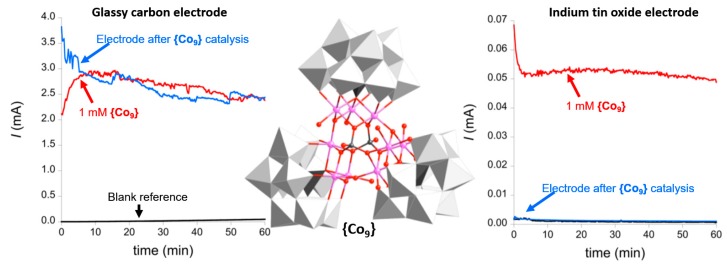
Left: Chronoamperometry of **{Co_9_}** (red) and the electrode immersed in a fresh, **{Co_9_}**-free electrolyte solution (red), showing that a deposited heterogeneous cobalt oxide/hydroxide is the active species under the given conditions. Center: Illustration of the structure of the Galán-Mascarós POM-WOC **{Co_9_}**, highlighting the central {Co_9_(H_2_O)_6_(OH)_3_(HPO_4_)_2_} stabilized by three polyoxotungstate ligands (grey polyhedra). Right: Chronoamperometry of **{Co_9_}** in the presence of bpy as Co(II) chelator (red) and the used electrode immersed in a fresh, **{Co_9_}**-free electrolyte solution (red), showing that no heterogeneous cobalt oxide/hydroxide is formed under the given conditions. Adapted from [28] with permission; copyright 2012 American Chemical Society.

**{Co_9_}:** As described above, the electrochemical WOC activity of **{Co_9_}** has been explored by Galán-Mascarós and colleagues. The authors performed a straight-forward electrochemical experiment to understand stability and degradation of **{Co_9_}** under electrocatalytic conditions (aqueous sodium phosphate buffer (50 mM) and NaNO_3_ as the electrolyte (1 M), pH 7, *E* = 1.41 V versus NHE). The system showed sustained oxidative catalytic currents (Figure 6, red trace) over 60 min. However, when the working electrode was removed from the solution, thoroughly washed and the same experiment was performed in a fresh, **{Co_9_}**-free electrolyte, virtually identical current traces were observed (Figure 6, blue trace). This strongly suggested that a heterogeneous catalyst had been deposited on the working electrode surface. Further analyses using scanning electron microscopy and energy-dispersive X-ray spectroscopy (SEM-EDX) indicated the presence of a cobalt oxide with traces of phosphate on the electrode surface, highlighting that, under the electrochemical conditions used, **{Co_9_}** can easily be converted into a solid-state cobalt oxide phase which is an active WOC. The authors hypothesized that Co(II) could be released from **{Co_9_}** as an intermediate to form the cobalt oxide films on the working electrode. To explore this, the authors performed the identical electrocatalytic experiments described above in the presence of 2,2’-bipyridine (bpy) as a Co(II) chelator to trap released Co^2+^ and prevent cobalt oxide deposition on the electrode. Note that in these experiments, indium tin oxide instead of glassy carbon was used as working electrode (Figure 6). In the presence of **{Co_9_}** in the electrolyte, the authors observed sustained catalytic oxidative currents. When the electrode was removed, washed and re-used in fresh, **{Co_9_}**-free electrolyte, no catalytic currents were observed (Figure 6). This finding demonstrates that the type of electrode strongly affects the deposition of heterogeneous metal oxide catalysts starting from polyoxometalate precursors. In addition, in the ITO experiment, the authors reported significantly lower currents (≈2 orders of magnitude compared with the glassy carbon experiments), which provides further evidence that different types of WOC were present in both experiments. Additionally, no cobalt oxide depositions were observed in the presence of bpy. This led the authors to conclude that under the study’s conditions (i.e., using bpy as the Co(II) scavenger and ITO working electrodes), **{Co_9_}** is an active homogeneous WOC [28].

## 6. Outlook and Future Directions

In the final section of this review, we provide the authors’ views on the major current challenges in the field, highlight promising future directions and emphasize recent emerging trends which could in future lead to new avenues for POM-WOC research.

In ground-breaking studies, Weinstock and colleagues have recently shown that POMs can be used as inorganic, stable ligands to stabilize colloidal metal oxide particles (TiO_2_, Fe_2_O_3_, etc.) in solution. This approach gives access to composite reactive colloids which show sustained WOC activity due to the presence of the POM stabilizers. Note that while the POMs in these systems are currently not considered the reactive WOC sites, they are nevertheless the sine qua non, without which the whole system would not be able to operate. Given the high technological potential of these composites for surface deposition, electrode immobilization or photoelectrode incorporation, a wide array of chemical modification possibilities has now become accessible and requires urgent research attention [102,103,104]. In addition, recent ground-breaking studies have shown that external stimuli, such as radio-frequency electromagnetic fields [105] or permanent magnetic fields [106], could be used to enhance the activities of WOCs. These and similar concepts could, in future, be used to fine-tune and optimize industrial water electrolysis.

While this review has been focused on molecular POM-WOC entities and their reactivity, a large field of research is also dealing with the use of POMs as precursors to nanostructured, solid-state metal oxides, most often for electrocatalytic WOCs. To this end, synthetic routes are being researched which enable the controlled conversion of the molecular POMs into active solid-state catalysts together with their stable deposition on suitable conductive substrates. Pioneering studies have, for example, explored the use of Keggin tungstates to deposit mixed cobalt copper tungsten oxides on metal electrodes, resulting in composites capable of oxygen evolution and hydrogen evolution [86]. Related studies have deposited POM-based metal oxides on carbon-based electrode materials; e.g., graphene for water oxidation catalysis [107].

Recently, pioneering studies have explored how POM-WOCs can be deposited and stabilized on the surfaces of light-absorbing semiconductors. Hill and colleagues used hematite (Fe_2_O_3_) photoelectrodes on which they deposited **{Ru_4_}** as an active catalyst. To prevent catalyst degradation and leaching under photoelectrochemical operation, a thin Al_2_O_3_ layer was deposited on top of the composite using atomic layer deposition. The authors observed that the Al_2_O_3_ film thickness effectively controlled catalyst stability and activity. Film thicknesses of 4 nm were optimum for WOC performance, whereas higher thicknesses resulted in loss of activity and lower thicknesses did not stabilize the catalyst sufficiently [108]. Related work by Solarska and colleagues used thin-film WO_3_ photoanodes decorated with POM ([H_3_PMo_12_O_40_])-coated gold nanoparticles for photoelectrochemical water splitting [109]. The authors reported that during water oxidation electrolysis, the POMs prevented photogenerated charge recombination, while the gold nanoparticles introduced plasmonic enhancements into the photoelectrode. Tuning of the system by incorporation of actual POM-WOCs instead of the Keggin species [H_3_PMo_12_O_40_] could further increase performance of this system, particularly when combined with the Hill-type Al_2_O_3_ coating approach. In summation, these photoelectrode fabrication principles can be used for a large variety of POM/substrate combinations and could have relevance for catalytic applications under harsh conditions beyond water oxidation, so more research work in the area is urgently required.

In summary, this review highlighted how polyoxometalate water oxidation catalysts can be deployed under homogeneous and heterogeneous electrochemical conditions. Clear reactivity and stability differences were discussed, and the role of the reaction conditions and heterogenization approaches with respect to POM reactivity were described, and insights gained by theory were discussed. Future directions of POM-WOC research, including novel immobilization and catalyst fabrication routes, were described together with emerging topics on the interface between molecular chemistry, materials chemistry and semiconductor science, which could lead to new materials design approaches for technologically viable POM-based water oxidation catalysts.
